# Prevalence of excessive screen time in hospitalized pediatric patients

**DOI:** 10.1016/j.jped.2025.02.005

**Published:** 2025-05-17

**Authors:** Guilherme Hoff Affeldt, Gleice Medeiros, Vanessa Vieira, Bruna Ziegler

**Affiliations:** a Hospital de Clínicas de Porto Alegre, Porto Alegre, RS, Brazil; b Universidade Federal do Rio Grande do Sul, Porto Alegre, RS, Brazil

**Keywords:** Screen, Screen time, Children, Functionality, Sedentary behavior

## Abstract

**Objective:**

This study aimed to understand the prevalence of screen time in hospitalized children and identify factors predicting excessive screen use during hospitalization.

**Methods:**

This cross-sectional quantitative study was conducted with patients from the Pediatric Inpatient Unit of a Brazilian hospital, from March 2022 to April 2023. A total of 260 children were included. Family members completed questionnaires about screen time during hospitalization and at home, as well as providing information on physical activity and functionality. Socioeconomic and demographic details were obtained from electronic records.

**Results:**

During hospitalization, children spent a median of 270 min per day on screens, significantly more than at home. Excessive screen time at home, better patient functionality, and lower caregiver education levels were significant predictors of excessive screen use during hospitalization.

**Conclusion:**

Excessive use of screen devices among hospitalized children, with only a minority adhering to the World Health Organization’s screen time recommendations. Key predictors of excessive screen use included high screen time at home, lower caregiver education levels, and preserved child functionality.

## Introduction

With the advent of the digital age, more and more children and adolescents are exposed to electronic devices.^1^ The daily routine of children and young people often involves various activities, with excessive screen use being prevalent in many cases, potentially impacting their health and development.^2^ In recent decades, access to technology has grown exponentially, with 87.4 % of 9th-grade students in Brazil owning a cell phone and 78 % of them spending at least two hours a day in front of the television.^3^

Excessive screen time (ST) can lead to many harms and health concerns for children and adolescents, such as adiposity, obesity, unhealthy diet,^4^ sleep disturbances,^5^ depressive symptoms,^6^ poor quality of life,^7^ and lower levels of physical activity.^8^

Excessive exposure to screen devices (SDs) is not restricted to the home environment. During periods of hospitalization, conditions such as the use of drains, probes, catheters, and the need for contact isolation can justify pediatric patients spending more time in front of screens. In the same study, it was revealed that participants between 4 months and 18 years were using screens in 80,3 % of their awake time.^9^ Although exposure to screens can be harmful to children, studies show that their use in hospital settings can benefit children by reducing anxiety levels and providing more relaxation during this period. Other studies with pediatric patients aged 7–12 years with cancer in a hospital context and undergoing chemotherapy showed that most of these patients prefer screen televisions and video games to play and feel better.^10^ It is important to show that screens could also be a solution sometimes to escape from loneliness, boredom emotional distress and promote academic skills during their period away from school.^9^ Given the harmful already reported from the excessive use of SDs, this study's main objective is to assess the prevalence of excessive screen time in hospitalized pediatric patients.

## Methods

This was a cross-sectional epidemiological study with prospective data collection. Participants were selected for convenience at the Pediatric Inpatient Unit of the Hospital de Clínicas de Porto Alegre (HCPA).

The study included pediatric patients aged 0 to 17 years with different diagnoses, of both sexes, admitted to the Pediatric Inpatient Unit from March 2022 to April 2023. Patients who had any impairment preventing them from using screens, who did not have family members present during hospitalization, or who did not remain in the hospital for at least 48 hours were excluded from the study. As part of an institutional strategy to reduce contagion among health professionals, patients diagnosed with COVID-19 were excluded.

Parents and participants were asked to sign an Informed Consent Form (ICF). After signing the informed consent form, personal and hospitalization-related information was collected using a questionnaire. This study was approved by the HCPA Research Ethics Committee, under opinion number 2019–0670 (CAAE 28515219300005327), in accordance with Resolution 466/2012 of the National Health Council of the HCPA.

Clinical and sociodemographic data was collected by consulting electronic medical records. Body mass index (BMI) was determined by calculating the ratio between weight (in kilograms) and height (in meters) squared. BMI was also expressed as a z score.^11^

Socioeconomic status was measured using the Brazilian economic classification criteria proposed by the Brazilian Association of Research Companies (ABEP). This is a questionnaire to analyze the household appliance parts and quantity that comprise the patient and family member’s home. By adding up all the items, the economic classification of each participant can be estimated on average, ranging from the lower class (R$ 900 average income) to the higher class (R$ 21,826 average income), considering the Brazilian values.^12^

The Functional Status Scale (FSS) is a scale that assesses functionality, developed for use with hospitalized children. The FSS results range from 6 to 30 points, and the higher the score, the greater the patient’s functional impairment.^13^

The International Physical Activity Questionnaire (IPAQ short version) is an instrument comprising questions related to the participants’ daily physical exercise habits and sedentary lifestyles. It indicates that the more time the interviewee spends doing physical activity on a daily basis, the more active and better classified the individual will be. Thus, the final result of this questionnaire allows the classification into: sedentary, insufficiently active, active, and very active.^14^ The questionnaire was scored for children over 6 years old.

Then, data was collected on the amount of time spent in front of screens at home and in the hospital, as well as the context of these activities. This information was collected using a questionnaire designed by the researchers, in which family members recorded how much time their children spent in front of screens at home and during hospitalization. They indicated which electronic devices the children used, as well as the reasons why screen devices (SDs) were offered, whether during excessive crying, boredom, or anxiety, among other reasons. In addition, the guardians filled in a memory diary in which they recorded all the activities the child had done with the device over the 24 hours of hospitalization. Patients were classified according to the time spent in front of the screens as “complies with the recommendation” or “does not comply with the recommendation.” The following criteria were used: children under 1 year old should not use screen devices^15^; children from 1 to 5 years old can spend a maximum of 1 hour/day in front of screens^15^; and children over 5 years old up to adolescence can spend a maximum of 2 hours/day.^15^ In addition to the diary, the researchers carried out direct observation of the patient’s behavior in bed at two different times, in different shifts, and with an interval of 6 hours between each one, thus increasing the reliability of the recall diary. The direct observation recorded which activity the child was doing at the time and whether they were using screen devices.

Lansky score is a scale that assesses the performance of the child’s activities of daily living, such as getting up and walking from a sitting position, for example. The scale ranges from 0 to 100 points, in which the higher the score, the better the child’s functionality. This scale is widely used for children with chronic illnesses and in palliative care and it is an important tool for guiding therapeutic conduct and evaluating the evolution of patients before and after therapies.^16^

### Statistical analysis

Data were expressed as absolute values (n) and percentages (%) or medians and interquartile ranges (II). The Shapiro-Wilk test was used to assess the normality of the variables and a graphical evaluation was performed.

McNemar’s test was used to compare the paired proportions of participants’ assessments at home versus in the hospital. A multivariate analysis was carried out with a robust estimate for the variances, adjusting each variable individually for gender and age, with the dependent variable being screen time. The data was stored in Microsoft Office Excel and analyzed using the SPSS program, version 18.0 (SPSS Inc., Chicago, IL, USA). The level of statistical significance adopted was 5 % (*p* < 0.05).

## Results

**Figure 1:**
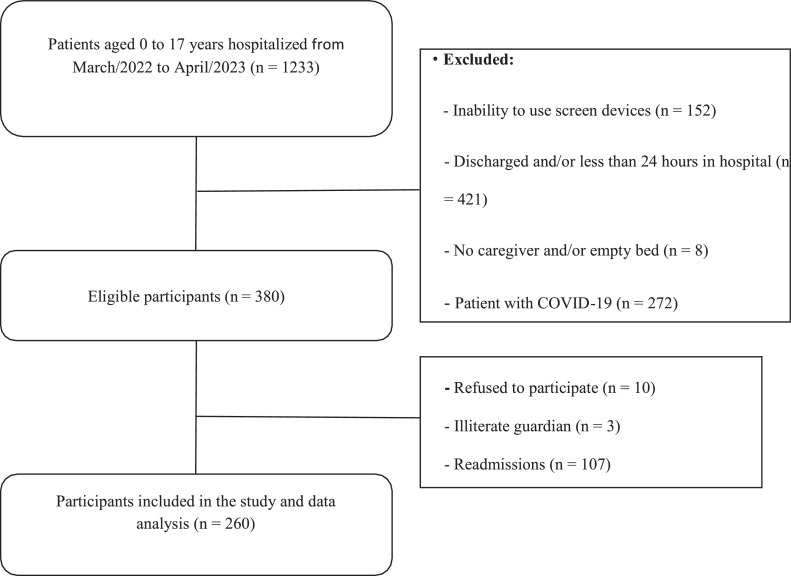
Participant selection flowchart.

From March 2022 to April 2023, 260 hospitalized children were included in the study (Figure 1). Of these, 61.5 % were male, with a median age of 5 (3–10) years of age and a BMI of 16.2 (14.9–19.4) Kg/m². Only 6.5 % of the participants were considered active. In this sample, 43 % of included participants were admitted to the hospital by oncological diseases, followed of 20 % by pneumological problems and 8,4 % by endocrinological disease. Other demographic characteristics of the study participants and their families can be seen in Table 1.

**Table 1:** Demographic characteristics of the study participants.

Variables	*n* = 260
Sex	
Male	160 (61.5 %)
Female	100 (38.5 %)
Type of disease that leads to admission	
Rheumatological	9 (3.46 %)
Dermatological	4 (1.53 %)
Genetic	6 (2.30 %)
Pneumological	52 (20 %)
Surgery	9 (3.46 %)
Hepatological	22 (8.46 %)
Endocrinological	19 (7.30 %)
Neurological	10 (3.84 %)
Oncological	112 (43 %)
Orthopedics	6 (2.30 %)
Urological	11 (4.23 %)
Ethnicity	
Caucasian	215 (82.6 %)
Non-Caucasian	45 (17.4 %)
Age (years)	5 (3–10)
BMI (Kg/m²)	6.2 (14.9–19.4)
BMI (Z score)	0.4 ± 1.5
Length of stay (days)	30 (10–76.5)
*Responsible person’s schooling*	
Primary education	90 (35 %)
High School	115 (44 %)
College/University	55 (21 %)
Socio-economic classification	
Lower class	92 (35.2 %)
Middle class	154 (59.4 %)
Upper class	14 (5.4 %)
IPAQ	
Active	17 (6.5 %)
Insufficiently active	46 (17.7 %)
Sedentary	50 (19.2 %)
ST diary in 24 h (minutes)	270 (125–413)
WHO classification	
Follow the recommendation	54 (20.8 %)
Not follow the recommendation	206 (79.2 %)
Lansky score (points)	80 (60–90)
*Functional Status Scale (points)*	7 (6–8)

BMI, body mass index; IPAQ, International Physical Activity Questionnaire; ST, screen time; WHO, World Health Organization.

Values expressed as number of cases (%) or median (interquartile range – IQR).

Around 217 (83.1 %) responsible family members reported that they realized the importance of regulating children’s screen time during hospitalization. However, 80.5 % of them reported that the use of SDs during this period can help relieve the distress imposed by hospitalization. Around 29.5 % of family members said that electronic media help children fall asleep and 20.3 % believe that they can help with feeding.

Table 2 shows the characteristics related to screen exposure factors and time spent in front of SDs during hospital and home environments. The authors verified that during hospitalization, participants spent more time watching television during the week (*p* = 0.020), and more time using portable TVs during the week (*p* < 0.001) and weekend (*p* < 0.001) when compared to at home. When family members were asked what situations led them to offer screen to the children, there was a higher proportion of pain symptoms (*p* < 0.001), crying or excessive complaining (*p* < 0.001), anxiety (*p* < 0.001), and boredom (*p* < 0.001) during the hospital stay compared to at home. At home, there was a higher prevalence of screens being offered in situations in which the responsible person was busy (*p* < 0.001) and for encouraging activities with educational videos (*p* = 0.016).

**Table 2:** Comparison between participants’ screen time during hospitalization and at home.

Variables (*n* = 260)	At home	At the hospital	*p*-value
Family member’s perception of ST use			
Much higher than desired	69 (26.4 %)	101 (38.7 %)	
Slightly above desired	117 (44.8 %)	65 (24.9 %)	<0.001
Adequate	64 (24.5 %)	75 (28.7 %)	
TV time on weekdays	120 (60–240)	180 (20–360)	0.020
Weekend TV time	180 (60–360)	180 (20–360)	0.549
Weekday portable SD time	120 (30–240)	180 (60–360)	<0.001
Situations in which the familiar offers the screen to the participants			
Busy	124 (47 %)	34 (13 %)	<0.001
Excessive crying or complaining	58 (22.2 %)	100 (38.3 %)	<0.001
Playing	45 (17.2 %)	43 (16.5 %)	0.885
Learning with educational videos	72 (27.6 %)	54 (20.7 %)	0.016
Pain	26 (10 %)	59 (22.6 %)	<0.001
Boredom	68 (26.1 %)	141 (54 %)	<0.001
Anxiety	44 (16.9 %)	83 (31.8 %)	<0.001

ST, screen time; SD, screen devices; TV, television.

Values expressed as number of cases (%) or median (interquartile range – IQR).

In the regression analysis, the authors observed that initial excessive screen time, better functionality according to the Lansky scale, and lower parental education were predictors of excessive screen time during hospitalization (Table 3).

**Table 3:** Multivariate linear regression considering the dependent variable screen time.

Variable	Multivariate	(ajusted for sex and age)	
	β	CI 95 %	*p*
FSS, points	−7.486	−21.418–6.446	0.292
Lansky Score, points	1.851	0.159–3.544	0.032
Length of stay, days	0.549	−0.356–1.455	0.235
BMI, Kg/m^2^	−1.44	−8.703–5.822	0.697
Screen time at home, minutes	0.256	0.081–0.0432	0.004
Physical activity, actives	−68.413	−219.93–83.104	0.376
Responsible education, high school	91.691	21.781–161.601	0.010

BMI, body mass index; FSS, functional status scale; CI, confidence interval.

## Discussion

In this study, the authors observed an excessive prevalence of screen time in children during hospitalization. Only a minimum number of included children followed the recommendation of the World Health Organization (WHO). In these analyses, it is possible to see that the amount of television time during the week in the hospital was significantly higher than that reported at home. Likewise, the amount of time participants spent on screen devices (tablets, cell phones, laptops) was significantly higher than that reported at home too, both during the week and at the weekend. As for the situations in which family members offered screen devices to their children, there was a higher proportion of device use due to crying or excessive complaining, boredom, pain, and anxiety during hospitalization. Whereas at home, there was a higher proportion of devices offered at times when caregivers were busy and to learn from educational videos.

In a children’s hospital in Greece, 546 children with an average age of 8.5 years were assessed for screen time at home and during hospitalization. As a result, the children spent 240 min a day on average in front of the television, about 30 min more than at home [17]. Another study of 96 hospitalized children showed that the average time spent in front of screens was 240 min during hospitalization and 120 to 200 min at home [9]. In Thailand, a study assessed 254 hospitalized children aged 44.5 months on average for screen exposure and screen time during hospitalization. This study's results showed that the median time spent in front of screens was 360 min, which is equivalent to more than a quarter of the day [18]. Other studies outside the hospital environment also suggest a high rate of screen time[19] and low adherence to the recommendations made by the world’s leading international public health bodies [20].

The present results showed that during hospitalization, family members offered screen devices in cases of crying, and times that the children felt anxious, to combat boredom and reduce pain. The study by Chaiseksamphan et al. [18] assessed the reasons why family members offered screens at the hospital to their children using a structured questionnaire. The study included 254 children with various types of illnesses, the most prevalent being hematological and infectious diseases. The median length of stay was 4 (3–12) days. Of the entire sample, 49 % of the children evaluated used screen devices when they were complaining or crying excessively, 94 % of times when they were bored and had no other activities during hospitalization, 58 % of cases when they had pain symptoms, and 77 % to play and relax. Excessive screens at home and in daily use can be linked to symptoms of depression and psychological distress [6,21]. During hospitalization, children may experience fear, sadness, and isolation, which can worsen these symptoms [22]. However, in a hospital setting, screens can also play a positive role by providing distraction, easing anxiety, and reducing the perception of pain, making the experience more bearable [22].

Despite the known harm, screen devices in the hospital environment can be important sources of distraction during medical procedures; and facilitate contact with family or friends outside the hospital, including peers, the community and schools [9]. The ability to connect with a child's school, community, and home helps normalize the experience, minimizing disruption to usual routines [9,10]. Digital technology, including interactive media and video games, moderately reduces pain and suffering in children undergoing painful procedures [9]. A meta-analysis revealed that digital distraction can lead to a significant decrease in self- and observer-reported pain and suffering during procedures such as venipuncture and dental treatments [23]. Additionally, interactive media has been shown to alleviate anxiety in pediatric patients, especially in environments like waiting rooms in rehabilitation hospitals, where it also enhances patient and family satisfaction [24].

In these findings, the best performance obtained by the Lansky score was a predictor of excessive screen time, curiously demonstrating that the more functionality the child had, the more time they spent on screens. Screen time is a global health problem, affecting healthy children of all ages, and the hypothesis is that participants with better functionality do not have any restrictions that hinder their use of screen media. On the other hand, children in a more serious condition may undergo a greater number of procedures and remain prostrate for longer, making it difficult for them to use SDs at times. The study by Dahgren et al. [25] evaluated 121 children and adolescents with 12.1 ± 1.5 years as the mean age to relate physical activity time to screen time in daily life. The results of this study showed that the most active children were also those who spent the longest on-screen devices. In the present study, family members with secondary and primary schooling spent more on screens during hospitalization, when compared to family members with higher education. In Spain, a study assessed the screen time of 1405 children aged 8 to 10 years on average in a city council program. In the intervention cities, the coordinator was selected from the community health department. Up to nine different community activities, such as familiar workshops about eating habits, screen time recommendations, and cooking techniques, were implemented in the intervention cities. This study showed that the mothers’ low schooling levels significantly increased the children’s screen time when compared to mothers with higher schooling levels [26]. Other authors have also noted the impact of the low educational level of family members on the increase in their children’s screen time [27,28].

Although the authors did not observe a negative association between good functionality and screen time, activities carried out in the in-hospital environment with music therapy and dance ensure an improvement in the pain and anxiety symptoms of children during hospitalization [29]. Stimulating the act of playing and exercising during hospitalization can maintain the child’s functionality, strength, and muscle tone, as well as help their motor development [30]. Creating ways to reduce exposure to SDs and promoting therapies that maintain functionality can be useful tools in combating the harmful effects of indiscriminate screen use, and reducing symptoms of anxiety and depression.

This is a differential by the evaluation of questionnaires on screen time by parents and especially by the acquisition of a 24-hour diary to measure the screen time of each participant included. The present sample consisted of children and adolescents aged 0 to 17 years, covering a wide age range and therefore heterogeneous. This study had some limitations. One of them refers to this sample, where it was only carried out in a single center, which limits knowledge among more children and from other hospitals in the city and in Brazil. The International Physical Activity Questionnaire was an instrument applied only to children over the age of 6, and the level of physical activity in younger children was not assessed. No data was collected on the quality of sleep of these children, which could be an interesting subject for future research. Lastly, the present sample consisted of children and adolescents aged 0 to 17 years, covering a wide age range and therefore heterogeneous. Otherwise, to emphasize some strengths, to date, there are few studies published that have managed to measure screen time so specifically and with such high reliability as this one. Another differential was the assessment of questionnaires on screen time by parents and especially the acquisition of a 24-hour diary to measure screen time for each participant included.

In conclusion, this study showed that only a minimum part of hospitalized children follow the screen time recommendations of the World Health Organization, spending a significant part of their hospitalized days in front of screens. Screen time during hospitalization was higher than at home and offered more often in moments when patients were with symptoms of pain, anxiety, and boredom at hospitalization. Excessive screen time at home, a low level of education, and preserved functionality were considered predictive factors of excessive screen time during hospitalization.

## Funding/Support

All phases of this study were supported by Financiamento e Incentivo à Pesquisa (Fipe/HCPA).

## Role of funder/sponsor (if any)

The Financiamento e Incentivo à Pesquisa (Fipe/HCPA) had no role in the design and conduct of the study.

## Authors’ contributions

All authors approved the final manuscript as submitted and agreed to be accountable for all aspects of the work.

## Conflicts of interest

The authors declare no conflicts of interest.
